# Prevalence, clinical characteristics, and hospital course of systemic sclerosis-associated pseudo-obstruction

**DOI:** 10.1007/s10067-025-07676-6

**Published:** 2025-10-01

**Authors:** Laura Ross, Lyman Lin, Dylan Hansen, Alannah Quinlivan, Wendy Stevens, Susanna Proudman, Jennifer Walker, Joanne Sahhar, Gene-Siew Ngian, Lauren Host, Mandana Nikpour, Chamara Basnayake

**Affiliations:** 1 Department of Medicine, University of Melbourne at St Vincent’s Hospital, Fitzroy, Australia; 2 Department of Rheumatology, St Vincent’s Hospital Melbourne, 41 Victoria Pde Fitzroy VIC, Fitzroy, 3065 Australia; 3 Department of Gastroenterology, St Vincent’s Hospital Melbourne, Fitzroy, Australia; 4 Rheumatology Unit, Royal Adelaide Hospital, Adelaide, Australia; 5 Discipline of Medicine, University of Adelaide, Adelaide, Australia; 6 Department of Rheumatology, Flinders Medical Centre, Bedford Park, Australia; 7 Department of Rheumatology, Monash Health, Clayton, Australia; 8 Department of Medicine, Monash University, Clayton, Australia; 9 Department of Rheumatology, Fiona Stanley Hospital, Murdoch, Australia; 10 School of Public Health, University of Sydney, Sydney, Australia; 11 Department of Rheumatology, Royal Prince Alfred Hospital, Sydney, Australia

**Keywords:** Enteric dysmotility, Gastrointestinal, Pseudo-obstruction, Systemic sclerosis

## Abstract

**Objective:**

Gastrointestinal involvement is almost universal in patients with systemic sclerosis (SSc). Enteric dysmotility, at its most severe, can present with pseudo-obstruction. In this study, we aimed to quantify the prevalence of SSc pseudo-obstruction and evaluate risk factors for its development. In a subgroup of patients requiring admission to hospital for acute episodes of pseudo-obstruction, we evaluated the clinical course and treatments administered.

**Methods:**

Using data from 1969 Australian Scleroderma Cohort Study (ASCS) participants, we performed multivariable logistic regression analysis to identify SSc-specific risk factors for pseudo-obstruction. Descriptive statistics were used to examine the clinical course of patients admitted with pseudo-obstruction at a single ASCS centre.

**Results:**

Pseudo-obstruction occurred uncommonly, affecting 70 (3.56%) ASCS participants. Records of 14 participants with a total of 39 admissions for acute pseudo-obstruction were identified. Pseudo-obstruction was associated with longer disease duration (OR 1.03, *p* = 0.03), bowel dysmotility (OR 4.51, *p* < 0.01), small intestinal bacterial overgrowth (OR 2.81, 95% CI (1.00–1.05), *p* < 0.01), and gastric antral vascular ectasia (OR 2.52, 95% CI 1.28–4.94, *p* < 0.01). Severe diarrhoea, as measured by the UCLA Gastrointestinal 2.0 questionnaire, was the only clinical symptom significantly associated with episodes of pseudo-obstruction (OR 3.39, 95% CI 1.56–7.38, *p* < 0.01). Opioids were more commonly prescribed in patients with pseudo-obstruction but were not significantly associated with pseudo-obstruction in multivariable analysis (OR 1.24, 95% CI 0.62–2.48, *p* = 0.54). Patients with a history of pseudo-obstruction were more likely to require enteral (4.29% vs. 0.21%, *p* < 0.01) or parenteral nutrition (7.14% vs. 0.16%, *p* < 0.01).

**Conclusion:**

Pseudo-obstruction is associated with other severe gastrointestinal manifestations and is associated with malnutrition in SSc patients. Future studies are required to assess the impact of treatment of SSc-associated enteric dysmotility to prevent progression to pseudo-obstruction.

**Table:** 

Key Points • Pseudo-obstruction is an uncommon manifestation of systemic sclerosis but frequently recurs and is associated with increased mortality. • Severe diarrhoea and long disease duration are associated with an increased risk of pseudo-obstruction. • Pseudo-obstruction occurs more commonly in patients with severe enteric dysmotility and gastric antral vascular ectasia (GAVE).

**Supplementary Information:**

The online version contains supplementary material available at 10.1007/s10067-025-07676-6.

## Introduction

The gastrointestinal tract is the most commonly involved internal organ system in systemic sclerosis (SSc) [1]. The exact pathogenic mechanism of gastrointestinal involvement is unknown, but early neural and inflammatory processes are thought to lead to smooth muscle atrophy, resulting in dysmotility, the most common manifestation of SSc gastrointestinal disease [1]. Upper gastrointestinal dysmotility commonly presents with symptoms of reflux, oropharyngeal and oesophageal dysphagia, nausea, and vomiting. Whilst occurring less frequently, intestinal and colonic dysmotility are associated with severe complications of small intestinal bacterial overgrowth (SIBO), acute and chronic pseudo-obstruction, malnutrition, and death.

Pseudo-obstruction can occur in cases of severe enteric dysmotility. Pseudo-obstruction is defined as evidence of intestinal or colonic dilatation with severe disruption of enteric motility, causing obstructive symptoms such as vomiting, abdominal pain and distension, and inability to pass faeces or flatus, in the absence of a mechanical obstruction. Pseudo-obstruction is a serious complication of SSc, with episodes of acute pseudo-obstruction associated with a 7% risk of in-hospital mortality [2]. Enteric dysmotility can be considered along a disease spectrum spanning irritable bowel syndrome (IBS) and enteric dysmotility (ED) through to chronic intestinal pseudo-obstruction [3, 4]. Whilst SSc is recognised as the commonest cause of secondary chronic intestinal pseudo-obstruction in adults [3, 4], there remains significant gaps in our understanding of the progression of enteric dysmotility in SSc, and in particular the clinical course of those patients with pseudo-obstruction.

In this study, we aimed to describe the prevalence and SSc features associated with pseudo-obstruction in the Australian Scleroderma Cohort Study (ASCS). Additionally, we performed a retrospective chart review of participants who presented to a single ASCS site (St Vincent’s Hospital Melbourne (SVHM)) for management of pseudo-obstruction. Data pertaining to presenting symptoms, investigations, treatment(s), and clinical outcomes were collected.

## Methods

The ASCS is a multi-centre prospective cohort study and all participants who fulfilled ACR/EULAR criteria for SSc [5] with a definable disease subclass [6] were included. Demographic and disease-related data were collected annually. Definitions of SSc-organ involvement are in Supplementary Index 1. Gastrointestinal symptoms were recorded (yes/no) annually. Medications, including nasogastric (NG), percutaneous endoscopic gastrostomy (PEG) feeding, or total parenteral nutrition (TPN), were recorded at each visit. Gastrointestinal investigations were performed at the discretion of the treating physician. Oesophageal and bowel dysmotility were recorded as suspected based on symptoms and confirmed if dysmotility was demonstrated by nuclear medicine or manometry testing. SIBO was recorded as present if participants received cyclical antibiotics to treat diarrhoea. Episodes of pseudo-obstruction (yes/no) were recorded at each study visit, defined by the treating physician based upon history and medical record review. From 2016, participants completed a Scleroderma Clinical Trials Consortium UCLA Gastrointestinal Tract 2.0 (GIT2.0) questionnaire annually. Severe diarrhoea and constipation were considered present if GIT2.0 component scores were > 1.25 and > 0.94, respectively [7].

### Case series

Medical record review of SVHM participants with a history of pseudo-obstruction was performed. Pseudo-obstruction was confirmed by two physicians (LL, CB) if vomiting, inability to open bowels and/or pass flatus, and confirmation of intestinal or colonic dilatation on imaging in the absence of a mechanical obstruction were all present. Data pertaining to presenting symptoms, investigation findings, treatment, and 30-day clinical outcomes were collected. SIBO prior to diagnosis of pseudo-obstruction and recent weight loss were recorded as present if documented in the medical record.

The ASCS is carried out in accordance with the *National Statement on Ethical Conduct in Research Involving Humans*. The study was approved by the Human Research Ethics Committee at SVHM (LRR 012/21), and written informed consent was provided before any data were collected.

### Statistical analysis

Data are presented as numbers (percentage) for categorical variables and median (interquartile range (IQR)) for continuous variables. Chi square test or Fisher’s exact test were used, as appropriate, to compare frequencies of categorical variables in those with and without pseudo-obstruction. Logistic regression analysis was performed to identify the associations of pseudo-obstruction (dependent variable). Variables of clinical relevance or statistically significant in univariable analysis were included in multivariable modelling. A separate multivariable model (model 2) was developed in patients with GIT2.0 questionnaire results available. Owing to the smaller population of this subgroup, only variables shown to be significant in multivariable modelling from model 1 were included in combination with severe diarrhoea. A time-dependent Cox-proportional hazard model that included sex, age, Scl70 status, pulmonary arterial hypertension, and interstitial lung disease was generated to evaluate the risk of death associated with pseudo-obstruction. All statistical analyses were performed using STATA 14.2 Software (StataCorp, College Station, TX, USA).

## Results

Of 1968 ASCS participants, 70 (3.56%) participants had a history of pseudo-obstruction. Forty-one participants reported an episode of pseudo-obstruction that occurred prior to ASCS recruitment. Participants were followed for a median 4.34 (1.45–8.59) years, and 18.61% (*n* = 366) of participants died (Table 1). Almost half (46.95%) of study participants completed $\ge$ 1 GIT2.0 questionnaire; 924 (46.95%) participants had a diarrhoea component scale available and 916 (46.54%) had a constipation component scale available.

**Table 1:** Population characteristics of 1968 systemic sclerosis patients of the Australian Scleroderma Cohort Study

	Overall population (*n* = 1968)	Pseudo-obstruction (*n* = 70)	No pseudo-obstruction (*n *= 1898)	*p* value^a^
Female (*n*, %)	1686 (85.67%)	61 (87.14%)	1625 (85.62%)	0.72
Diffuse cutaneous involvement (*n*, %)	498 (25.30%)	26 (37.14%)	472 (24.87%)	0.02
Age at disease onset (years, median (IQR))	47.36 (36.77–57.62)	45.79 (33.11–53.63)	47.38 (36.98–57.72)	0.15
Disease duration at recruitment (years, median (IQR))	7.20 (2.52–15.69)	10.33 (5.20–17.62)	7.03 (2.46–15.62)	0.04
Disease duration < 5 years at recruitment (*n*, %)	727 (39.38%)	16 (24.62%)	711 (39.92%)	0.01
Death during follow-up (*n*, %)	366 (18.61%)	17 (24.29%)	349 (18.40%)	0.21
ANA positive (*n*, %)	1826 (92.78%)	62 (88.57%)	1764 (92.94%)	0.07
Centromere positive (*n*, %)	882 (44.82%)	32 (46.38%)	850 (46.58%)	0.97
Scl70 positive (*n*, %)	279 (14.18%)	6 (8.57%)	273 (15.22%)	0.16
RNA polymerase III positive^b^ (*n*, %)	180 (13.66%)	8 (11.43%)	172 (9.06%)	0.29
*SSc gastrointestinal involvement*				
Reflux oesophagitis (*n*, %)	954 (48.48%)	50 (71.43%)	904 (47.63%)	< 0.01
Oesophageal dysmotility (*n*, %)	232 (11.79%)	17 (24.29%)	215 (11.33%)	< 0.01
GAVE (*n*, %)	173 (8.79%)	13 (18.57%)	160 (8.43%)	< 0.01
Bowel dysmotility (*n*, %)	92 (4.67%)	15 (21.43%)	77 (4.06%)	< 0.01
SIBO (*n*, %)	156 (7.93%)	19 (27.14%)	137 (7.22%)	< 0.01
Severe diarrhoea^c^ (*n*, %)	212 (22.94%)	17 (51.52%)	195 (21.86%)	< 0.01
Severe constipation^d^ (*n*, %)	272 (29.66%)	14 (42.42%)	258 (29.19%)	0.10
Faecal incontinence (*n*, %)	636 (32.32%)	42 (60.00%)	594 (31.30%)	< 0.01
Annual weight loss$\ge$10% (*n*, %)	328 (21.90%)	16 (26.23%)	312 (21.71%)	0.40
NG or PEG feeding (*n*, %)	7 (0.39%)	3 (4.29%)	4 (0.21%)	< 0.01
TPN (*n*, %)	8 (0.44%)	5 (7.14%)	3 (0.16%)	< 0.01
*Other disease manifestations*				
Digital ulcers (*n*, %)	1041 (52.90%)	43 (61.43%)	998 (52.58%)	0.15
Interstitial lung disease (*n*, %)	550 (27.95%)	26 (37.14%)	524 (27.60%)	0.20
Pulmonary arterial hypertension (*n*, %)	235 (11.94%)	8 (11.43%)	227 (11.96%)	0.89
Scleroderma renal crisis (*n*, %)	72 (3.66%)	3 (4.29%)	69 (3.64%)	0.78
Myositis (*n*, %)	139 (7.06%)	5 (7.14%)	134 (7.06%)	0.98
*Treatment*				
Calcium channel antagonists (*n*, %)	1291 (65.63%)	45 (64.29%)	1246 (65.65%)	0.62
Prednisolone (*n*, %)	901 (45.78%)	33 (47.14%)	868 (45.73%)	0.82
Mycophenolate (*n*, %)	276 (14.02%)	10 (14.29%)	266 (14.01%)	0.95
Opioids (*n*, %)	235 (11.94%)	14 (20.00%)	221 (11.64%)	0.03
Promotility agents (*n*, %)	321 (16.32%)	30 (42.86%)	291 (15.34%)	< 0.01

^a^Comparison between those with and without history of pseudo-obstruction

^b^Of 1318 (66.97%) of patients who had RNA polymerase III testing

^c^924 participants who completed$\ge$1 SCTC UCLA GIT 2.0 diarrhoea component questionnaire

^d^916 participants who completed$\ge$1 SCTC UCLA GIT 2.0 constipation component questionnaire

*GAVE* gastric antrum vascular ectasia, *IQR* inter-quartile range, *NG* nasogastric, *PEG* percutaneous endoscopic gastronomy, *Scl70* anti-topoisomerase I, *SIBO* small intestinal bacterial overgrowth, *SSc* systemic sclerosis, *TPN* total parenteral nutrition

In those with a history of pseudo-obstruction, reflux (95.71% vs. 85.82%, *p* = 0.02), vomiting (16.18% vs. 6.71%, *p* < 0.01), dysphagia (70.00% vs. 53.76%, *p* < 0.01), and severe diarrhoea (51.52% vs. 22.08%, *p* < 0.01) were more common compared to those without pseudo-obstruction. Participants with pseudo-obstruction were more likely to be exposed to opioids (20.00% vs. 11.64%, *p* = 0.03). Participants with a history of pseudo-obstruction were more likely to have SIBO (27.14% vs. 7.22%, *p* < 0.01), receive promotility agents (42.86% vs. 15.34%, *p* < 0.01), NG/PEG feeding (4.29% vs. 0.21%, *p* < 0.01), or TPN (7.14% vs. 0.16%, *p* < 0.01). There was no difference in the frequency of significant weight loss between those with or without pseudo-obstruction (26.23% vs. 21.71%, *p* = 0.43) (Table 1). A history of pseudo-obstruction was associated with an increased risk of death (HR 1.97 (95% CI 1.13–3.39), *p* = 0.02) (Supplementary Index 2).

### Clinical associations of pseudo-obstruction

**Fig. 1:**
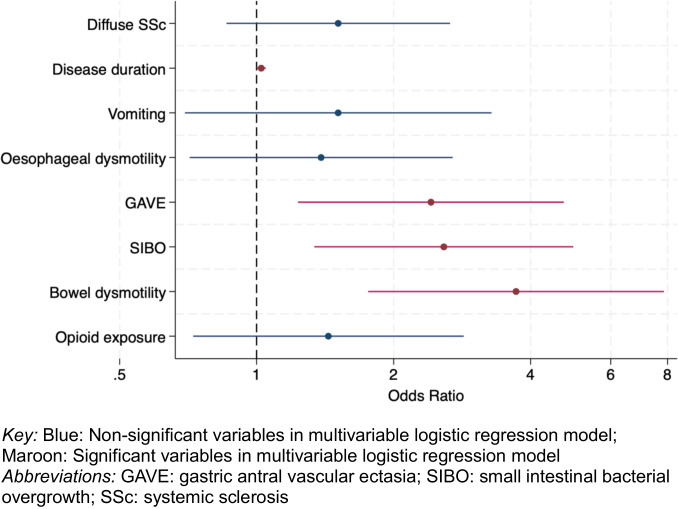
Forest plot of risk factors for pseudo-obstruction

Longer disease duration was associated with an increased risk of pseudo-obstruction (OR 1.03, 95% CI 1.00–1.05, *p* = 0.03), as was severe stomach and intestinal involvement (GAVE: OR 2.52, 95% CI 1.28–4.94, *p* < 0.01; SIBO: OR 2.81, 95% CI 1.46–5.40, *p* < 0.01; bowel dysmotility: OR 4.51, 95% CI 2.12–9.58, *p* < 0.01) (Table 2 and Fig. 1, univariable analysis in Supplementary Index 3). Despite being more commonly prescribed in those with pseudo-obstruction, opioid exposure was not associated with pseudo-obstruction (OR 1.24, 95% CI 0.62–2.48, *p* = 0.54). One hundred and two (5.18%) of patients used opioids at two or more consecutive visits, and similarly, this was not significantly associated with pseudo-obstruction (data not shown). Multivariable modelling that included only disease duration and other gastrointestinal involvement showed that symptoms of severe diarrhoea strongly predicted the presence of pseudo-obstruction (OR 3.39, 95% CI 1.56–7.38, *p* < 0.01).

**Table 2:** Multivariable logistic regression model of associations of pseudo-obstruction in Australian Scleroderma Cohort Study participants

Model 1			Model 2		
Overall cohort *n* = 1989			Patients with GIT 2.0 questionnaire available *n* = 924		
Clinical variable	OR (95% CI)	*p* value	Clinical variable	OR (95% Ci)	*p* value
Diffuse	1.54 (0.88–2.73)	0.13			
Disease duration	1.03 (1.00–1.05)	0.03	Disease duration	1.03 (1.00–1.07)	0.03
Vomiting^a^	1.34 (0.60–2.97)	0.47			
Oesophageal dysmotility	1.40 (0.71–2.74)	0.33			
GAVE	2.52 (1.28–4.94)	0.01	GAVE	2.92 (1.12–7.64)	0.03
SIBO	2.81 (1.46–5.40)	< 0.01	SIBO	7.23 (3.15–16.61)	< 0.01
Bowel dysmotility	4.51 (2.12–9.58)	< 0.01		2.43 (0.69–8.59)	0.17
Opioid exposure	1.24 (0.62–2.48)	0.54			
			Severe diarrhoea	3.39 (1.56–7.38)	< 0.01

^a^Patient-reported vomiting of at least once weekly

*CI* confidence interval, *GAVE* gastric antrum vascular ectasia, *GIT 2.0* SCTC UCLA GIT 2.0 questionnaire, *OR* odds ratio, *SIBO* small intestinal bacterial overgrowth

### Case series

Twenty-two patients at SVHM recorded an episode of pseudo-obstruction, representing 31% of all ASCS participants with a history of pseudo-obstruction. Medical records were available for 14 (63.64%) patients. Collectively, these patients had 39 pseudo-obstruction admissions (Table 3). Seven patients (50%) had multiple presentations of pseudo-obstruction, with the highest number being 15. Twelve patients (85.71%) had confirmed gastrointestinal dysmotility prior to the first presentation of pseudo-obstruction, with bowel dysmotility (*n* = 9, 64.29%) more common than oesophageal dysmotility (*n* = 4, 28.57%). Almost three quarters (71.43%) of patients admitted for management of pseudo-obstruction had dcSSc. RNA polymerase III (RNAPIII) autoantibodies were observed in 42.86% of patients, which is significantly more prevalent than in the general SVHM SSc cohort (42.86% vs. 16.63%, *p* < 0.01). Patients were commonly on treatment for enteric dysmotility (cyclical antibiotics (*n* = 6, 42.86%); promotility agents (*n* = 7, 50.00%)).

**Table 3:** Characteristics of case series of 17 patients admitted to a single Australian Scleroderma Cohort Study centre for inpatient management of pseudo-obstruction

Pt	Age^a^ (yrs)	Sex	Number episodes	Subtype	Ab	Confirmed SSc GI manifestations prior to pseudo-obstruction				Prescribed medications used prior to episode pseudo-obstruction			Clinical presentation at 1 st pseudo-obstruction		Imaging findings at 1 st pseudo-obstruction		LOS (days)^b^	Treatment^c^
						Oesoph dysmotility	GAVE	SIBO	Bowel dysmotility	Cyclical abx	Promotility agents	Opioid	Symptoms	BNO	AXR	CT Abdo		
1	62	F	1	Diffuse	ACA RNAP III	Y	Y				Y	Y	Nausea Vomiting Abdo pain Constipation	N	Faecal impaction, dilated duodenum		24	NBM IVF Elect Abx NGT Promot Gastrograf
2	74	F	1	Limited	RNAP III	Y							Nausea Vomiting Reduced oral intake Abdo pain Constipation	Y	SB: dilated	SBO	13	NBM IVF Elect NGT Promot Gastrograf
3	62	M	1	Diffuse	RNAP III				Y				Reduced oral intake Abdo pain Constipation	Y	SB: dilated Air fluid levels		2	Observed
4	61	F	3^d^	Diffuse	ANA (speck)		Y	Y	Y	Y			Nausea Vomiting Reduced oral intake Diarrhoea Weight loss	N	SB: dilated	SB: dilatation proximal and ileal SB loops Bunching plicae circulare	26	IVF Elect NGT NG feed TPN Gastrograf
5	64	F	1	Diffuse	RNAP III								Reduced oral intake Weight loss	N		SB: dilatation LB: dilatation	46	NBM IVF Elect Promot Gastrograf
6	70	F	2	Diffuse	ANA (nuc/speck)	Y		Y	Y	Y	Y		Nausea Vomiting Reduced oral intake Abdo pain Diarrhoea Weight loss	N	SB: dilated Air fluid levels		6–28	NBM (1) IVF (2) Abx (2) NGT (1) TPN (1) Promot (1)
7	53	F	5	Limited	ACA			Y	Y	Y	Y		Nausea Vomiting Abdo pain Constipation	Y	LB: dilated		8–12	NBM (1) IVF (4) Elect (3) Abx (3) Promot (5) Gastrograf (1)
8	79	F	2	Diffuse	Scl70				Y				Nausea Vomiting Reduced oral intake Abdo pain Constipation Incontinence	Y	LB: dilated	Pan-colonic faecal loading	2–20	NBM (1) IVF (2) Elect (1) Promot (2) Gastrograf (1)
9	53	M	1	Diffuse	RNAP III	Y	Y	Y		Y	Y		Nausea Vomiting Reduced oral intake Diarrhoea Weight loss	N		SB: dilated	9	IVF
10	77	F	1	Limited	ACA				Y				Nausea Vomiting Abdo pain Diarrhoea	N		SBO with mesenteric ischaemia	7	NBM IVF Elect Abx NGT Surgery
11	46	F	2	Diffuse	ANA (speck)						Y		Nausea Vomiting Reduced oral intake Abdo pain	N	SB: dilated LB: dilated	SB: dilated	8–16	NBM (1) IVF (2) Abx (2) NGT (1) Promot (2)
12	61	F	5	Diffuse	ANA (speck)		Y	Y	Y	Y	Y		Nausea Vomiting Reduced oral intake Diarrhoea Weight loss	N	SB: dilated	SB: dilatation proximal & ileal SB loops Bunching plicae circulare	2–70	NBM (1) IVF (4) Elect (4) Abx (3) NGT (2) NG feed (1) TPN (4) Promot (1) Gastrograf (2)
13	62	M	1	Diffuse	RNAP III				Y				Nausea Vomiting Abdo pain Diarrhoea	N	SB: dilated Air fluid levels		7	NBM IVF Elect NGT Promot Gastrograf
14	68	F	15	Limited	ANA (speck)			Y	Y	Y	Y		Nausea Vomiting Abdo pain Diarrhoea	N	SB: dilated		2–15	NBM (9) IVF (13) Elect (13) Abx (11) NGT (4) Promot (4) Gastrograf (2)

^a^Age at first pseudo-obstruction presentation

^b^Range if multiple pseudo-obstruction admissions

^c^Treatments prescribed; frequency of use recorded in brackets in cases of multiple pseudo-obstruction admission

^d^First two admissions at another institution; details of these admissions not recorded in this table

*Ab* antibodies, *Abdo* abdominal, *Abx* antibiotics, *ANA* anti-nuclear antibody, *AXR* abdominal x-ray, *BNO* bowels not opening, *CT Abdo* computed tomography of the abdomen and pelvis, *Elect* intravenous electrolyte replacement, *GAVE* gastric antrum vascular ectasia, *Gastrograf* gastrograffin, *GI* gastrointestinal, *IVF* intravenous fluids, *LB* large bowel, *LOS* length of stay, *N* no, *NBM* nil by mouth, *NG feed* nasogastric feeding, *NGT* nasogastric tube – for decompression, *nuc* nucleolar ANA pattern, *Oesoph* oesophageal, *Promot* promotility agents, *Pt* patient, *SB* small intestine, *SBO* small bowel obstruction, *SIBO* small intestinal bacterial overgrowth, *SSc* systemic sclerosis, *speck* speckled ANA pattern, *TPN* total parenteral nutrition, *Y* yes

Treatment of pseudo-obstruction was highly variable. Intravenous fluids (*n* = 13, 92.86%), electrolyte replacement (*n* = 10, 71.43%), promotility agents (*n* = 10, 62.50%), antibiotics (*n* = 7, 50.00%), and gastrograffin (*n* = 9, 64.29%) were the most prescribed treatments. Only one patient had surgical intervention. Two patients (12.50%) died during a pseudo-obstruction admission; one of acute pseudo-obstruction and the second patient died of sepsis.

## Discussion

Pseudo-obstruction is an infrequent but severe SSc manifestation, observed in 3.56% of ASCS participants. Acute pseudo-obstruction was associated with a high in-hospital mortality rate, and pseudo-obstruction at any time was associated with an increased all-cause mortality risk. Pseudo-obstruction was associated with longer disease duration and other gastrointestinal manifestations such as severe diarrhoea, SIBO, enteric dysmotility, and GAVE. The association with GAVE has not previously been described and is notable because of the hypothesised contribution of microvascular injury to the development of SSc gastrointestinal disease [8]. Multiple processes of microvasculopathy, neuronal changes, circular muscle layer fibrosis, and smooth muscle atrophy are all documented and potentially occur in parallel throughout the gastrointestinal tract [1, 8], all of which may contribute to the development of severe dysmotility.

It is notable that the autoantibody associations of pseudo-obstruction in the ASCS were distinct from other cohorts [9]. We observed a high frequency of RNAPIII positive patients in our case series. In the overall ASCS population, we did not find an association between pseudo-obstruction and myositis and male sex, as has been previously reported [9, 10]. However, consistent with previous studies, we observed an association of pseudo-obstruction with dcSSc. Geographic and racial variability in the genotype and phenotype of SSc patients has been observed [11, 12]. The differences in SSc phenotype of those with pseudo-obstruction in the ASCS may reflect genotypic differences in the Australian SSc cohort. Racial differences and differences in autoantibody profile of the Australian cohort may account for some of the differences reported here compared to previous studies of SSc pseudo-obstruction [9, 10].

Whether enteric dysmotility in SSc is reversible and progression to pseudo-obstruction is preventable is unclear. We have observed that enteric dysmotility confirmed by nuclear medicine studies or manometry is far more common than pseudo-obstruction, indicating that not all patients with dysmotility progress to pseudo-obstruction. Small studies have consistently demonstrated delayed enteric transit time in SSc [13], and delayed colonic transit time has been associated with pseudo-obstruction and malabsorption [14]. However, future work is needed to evaluate SSc enteric dysmotility in the absence of symptoms to better appreciate the natural history of this disease manifestation and identify patients at high risk of progression to pseudo-obstruction, including whether specific anatomical sites of gastrointestinal dysmotility can be used to risk-stratify patients.

In our case series, 50% of patients had multiple presentations with pseudo-obstruction. This is higher than the 27% recurrence rate reported from two US university centres [15]. However, both studies highlight that acute pseudo-obstruction is frequently not an isolated event. Our study population was too small to ascertain whether there are specific SSc features that might place a patient at increased risk of recurrent disease. Multiple presentations of acute pseudo-obstruction raise the possibility of development of chronic intestinal pseudo-obstruction, with the attendant risks of malnutrition due to intestinal failure and death [16, 17]. Risk of malnutrition is further exacerbated if patients restrict oral intake to manage lower gastrointestinal tract symptoms [3]. Our results do not show that SSc patients with pseudo-obstruction lose weight with any greater frequency than other SSc patients. However, the need for nutritional support was significantly higher in those with pseudo-obstruction; therefore, weight loss may be attenuated by enteral and parenteral feeding. The lack of association between pseudo-obstruction and weight loss may be accounted for by the multi-factorial causes of weight loss in SSc rather than pseudo-obstruction itself not being an important cause of weight loss.

Whilst ASCS patients with pseudo-obstruction were more likely to have been exposed to opioids, their use was not predictive of the development of pseudo-obstruction. We were unable to analyse the effect of timing of opioid exposure and pseudo-obstruction presentation. The ASCS does not collect type of opioid prescribed or medication dosage, so it was not possible to examine the effect of total opioid dose on risk of pseudo-obstruction. It is possible that the dose and duration of opioid use may influence risk of pseudo-obstruction, and this should be examined in future studies. Given the known reduction in intestinal secretion and motility delay from opioid receptor agonism [18], we would still advocate cautious use of opioid medications in individuals with known enteric dysmotility. However, our results do highlight that when pseudo-obstruction occurs in SSc, it commonly does so in patients with no previous opioid exposure.

Identification of effective treatments for SSc gastrointestinal manifestations was an identified area of high unmet need in the recent EULAR SSc treatment recommendations [19]. No specific guidance about the treatment of SSc pseudo-obstruction was included in these recommendations, and use of promotility medication for symptomatic gastrointestinal dysmotility was conditionally recommended [19]. There are few SSc specific data to guide decisions about the treatment of SSc enteric dysmotility, and there are no known medications which reduce the frequency of pseudo-obstruction in non-SSc chronic intestinal pseudo-obstruction. Fluid, electrolyte, and nutritional balance are key aspects of the treatment of pseudo-obstruction [20]. Nutritional support is frequently required in patients with chronic pseudo-obstruction, including enteral and or parenteral nutritional supplementation when oral intake is poorly tolerated or inadequate to meet nutritional needs [21]. Treatment of SIBO is recommended because of the recognised association between bacterial overgrowth and pseudo-obstruction, and the increased risk of malnutrition associated with SIBO [22]. Non-opioid analgesia should be used when patients have abdominal pain, with consideration of agents such as gabapentinoids or tricyclic antidepressants [4, 21]. Promotility medications may improve symptom control and enable greater oral nutritional intake; however, there is no data showing that medical management can alter the natural history of enteric dysmotility [4, 20]. Agents such as pyridostigmine (acetyl cholinesterase inhibitor) and prucalopride (selective high-affinity 5-hydroxytryptamine 4 receptor antagonist) can be considered in patients with confirmed dysmotility and, or pseudo-obstruction [21, 23].

Our study is not without limitations. The diagnosis of pseudo-obstruction in the ASCS is recorded as present, according to physician diagnosis with no reference to clinical or imaging abnormalities, and the majority of ASCS participants who reported pseudo-obstruction had a first episode prior to recruitment. The ASCS database does not record gastrointestinal investigation results, meaning it was not possible to verify the diagnosis of pseudo-obstruction with reference to imaging abnormalities in the overall cohort. Diagnostic verification with reference to presenting symptoms and imaging could only be performed in the subgroup of patients presented in the case series. Imaging data and detailed symptom and management data were not available for those participants who presented at sites other than SVHM. Future studies of pseudo-obstruction should include imaging findings to improve the diagnostic certainty of this important disease manifestation and enable the standardisation of the classification of episodes of SSc pseudo-obstruction. The small number of verified episodes of pseudo-obstruction included means that only cautious conclusions can be drawn from this data. The clinical characteristics and disease course of these hospitalised patients may not necessarily reflect the natural history of pseudo-obstruction in the broader SSc patient population. Larger studies, with more systematic application of standardised, serial investigations are required to better understand the natural history of SSc dysmotility and to identify opportunities for effective therapeutic intervention.

Many patients in the ASCS have long-standing SSc, meaning study results are at risk of survivor bias. In particular, this may affect survival analyses performed using ASCS data. Patients with aggressive disease who die quickly as a result of their SSc are likely under-represented in this cohort. These study findings may not be applicable to patients with early-onset and rapidly progressive SSc. There is no protocolised investigation of the gastrointestinal tract in the ASCS. Participants are more likely to undergo investigation when presenting with gastrointestinal symptoms; therefore, the risk of pseudo-obstruction associated with imaging abnormalities or endoscopic findings estimated using ASCS data may be overestimated. The absence of standardised, repeated investigations precludes any assessment of the progression of enteric dysmotility in the ASCS. The statistical power of any analyses is limited by the infrequent occurrence of pseudo-obstruction, and the differences in disease associations observed in this study as compared to previously published reports may be accounted for by limitations of statistical power in addition to the potential genetic variation of the ASCS cohort compared to other cohorts internationally.

Despite these limitations, we have documented the prevalence of pseudo-obstruction in a large SSc cohort and highlighted important clinical associations to identify a patient phenotype at increased risk of pseudo-obstruction. Given the significant morbidity and mortality associated with pseudo-obstruction, our results suggest that patients with long-standing disease presenting with severe diarrhoea should be assessed for the presence of pseudo-obstruction. Further research is required to assess whether intervention prior to the onset of pseudo-obstruction can prevent progression of gastrointestinal dysmotility.

## Supplementary Information

Below is the link to the electronic supplementary material.

ESM 1 Supplementary Material 1 (DOCX 26.6 KB)

## Data Availability

Data is available on reasonable request, subject to Human Research Ethics Committee review.
