# Supplementary material for: Prevalence, clinical characteristics, and hospital course of systemic sclerosis-associated pseudo-obstruction
Source: Clin Rheumatol. 2025 Oct 1;44(11):4541–51. doi: 10.1007/s10067-025-07676-6 (PMC12568857; doi:10.1007/s10067-025-07676-6)
Supplement: Supplementary file 1 — Supplementary Material 1 (DOCX 26.6 KB) [file 10067_2025_7676_MOESM1_ESM.docx]

**Supplementary Index 1: Australian Scleroderma Cohort Study organ involvement definitions**

Disease duration was defined as time from the onset of first non-Raynaud’s phenomenon SSc manifestation. including reflux (heartburn), dysphagia, post-prandial bloating, vomiting, diarrhoea, constipation, faecal incontinence, and unintended weight loss were recorded (yes/no). Participants were screened annually for pulmonary hypertension and interstitial lung disease. All participants had annual respiratory function tests (RFTs) and transthoracic echocardiography (TTE) to screen for interstitial lung disease (ILD) and pulmonary hypertension. Participants were considered to have ILD if typical lung parenchymal changes were observed on high resolution computed tomography of the chest (HRCT). Individuals were referred for HRCT at the discretion of the treating physician, based on abnormal examination or investigation findings. Pulmonary arterial hypertension (PAH) was confirmed if a mean pulmonary artery pressure $\geq$20mmHg, pulmonary arterial wedge pressure $\leq$15mmHg and pulmonary vascular resistance $\geq$3 Wood units were confirmed by right heart catheterisation.^8^ Digital ulcers were considered present if the patient had ever reported a history of digital tip ulceration or a SSc digital ulcer had been observed on clinical examination. Myositis was determined by physician assessment based upon the presence of clinical features of weakness, elevated creatine kinase, typical magnetic resonance imaging or electromyography findings, or positive muscle biopsy. Reflux oesophagitis and gastric antral vascular ectasia (GAVE) were confirmed by endoscopy.

**Supplementary Index 2: Multivariable Cox regression model of survival**

| **Variable** | **Hazard Ratio**  **(95% CI)** | **p value** |
| --- | --- | --- |
| Pseudo-obstruction | 1.97  (1.14 – 3.39) | p=0.015 |
| Female | 0.62  (0.48 – 0.81) | p<0.001 |
| Age | 1.06  (1.04 – 1.07) | p<0.001 |
| Scl70 | 1.08  (0.78 – 1.50) | p=0.630 |
| PAH | 4.67  (3.59 – 6.07) | p<0.001 |
| ILD | 2.26  (1.80 – 2.84) | p<0.001 |

*Abbreviations:* ILD: interstitial lung disease; PAH: pulmonary arterial hypertension

**Supplementary Index 3: Univariable logistic regression analysis – associations of pseudo-obstruction**

|  | **OR**  **(95% CI)** | **p value** |
| --- | --- | --- |
| Age at disease onset | 0.99  (0.97-1.00) | 0.11 |
| Disease duration at recruitment | 1.03  (1.01-1.05) | 0.01 |
| Female | 1.16  (0.57-2.37) | 0.68 |
| Diffuse | 1.79  (1.09-2.94) | 0.02 |
| Centromere | 0.99  (0.61-1.60) | 0.96 |
| Scl70 positive | 0.54  (0.23-1.26) | 0.15 |
| RNA polymerase III | 1.48  (0.67-3.26) | 0.33 |
| Digital ulcers | 1.42  (0.87-2.32) | 0.16 |
| Interstitial lung disease | 1.71  (0.79-3.70) | 0.17 |
| Pulmonary arterial hypertension | 0.93  (0.44-1.96) | 0.85 |
| Myositis | 0.96  (0.38-2.43) | 0.93 |
| Reflux | 0.98  (0.91-1.06) | 0.59 |
| Dysphagia | 1.00  (0.99-1.01) | 0.63 |
| Vomiting* | 2.68  (1.37-5.26) | <0.01 |
| Oesophageal dysmotility | 2.52  (1.43-4.44) | <0.01 |
| GAVE | 2.42  (1.29-4.52) | 0.01 |
| Bowel dysmotility | 7.71  (4.12-14.44) | <0.01 |
| SIBO** | 4.94  (2.83-8.64) | <0.01 |
| Severe diarrhoea | 3.75  (1.86-7.56) | <0.01 |
| Severe constipation | 1.76  (0.87-3.57) | 0.12 |
| >10% weight loss | 1.26  (0.71-2.27) | 0.43 |
| Prednisolone | 1.08  (0.67-1.75) | 0.75 |
| Calcium channel antagonist | 0.88  (0.54-1.45) | 0.62 |
| Mycophenolate | 0.96  (0.48-1.90) | 0.90 |
| Opioids | 1.72  (0.94-3.15) | 0.08 |

*patient-reported vomiting of at least once weekly

**defined as use of cyclical antibiotics for treatment of bacterial overgrowth

*Abbreviations:* CI: confidence interval; GAVE: gastric antrum vascular ectasia; GI: gastrointestinal; Scl70: anti-topoisomerase I; SIBO: small intestinal bacterial overgrowth
